# Development of a cost-effective medium for *Photorhabdus temperata* bioinsecticide production from wastewater and exploration of performance kinetic

**DOI:** 10.1038/s41598-020-80773-5

**Published:** 2021-01-12

**Authors:** Sahar Keskes, Wafa Jallouli, Imen Ben Atitallah, Fatma Driss, Emna Sahli, Mohamed Chamkha, Slim Tounsi

**Affiliations:** 1 Laboratory of Biopesticides, Centre of Biotechnology of Sfax, Sfax University, P.O. Box ‘1177’, 3018 Sfax, Tunisia; 2 Laboratory of Enzyme Engineering and Microbiology, National School of Engineering of Sfax (ENIS), Sfax University, BP 1173, 3038 Sfax, Tunisia; 3 Analysis Laboratory, Centre of Biotechnology of Sfax, Sfax University, P.O. Box ‘1177’, 3018 Sfax, Tunisia; 4 Laboratory of Environmental Bioprocesses, Centre of Biotechnology of Sfax, Sfax University, P.O. Box ‘1177’, 3018 Sfax, Tunisia

**Keywords:** Biotechnology, Microbiology

## Abstract

This study investigates the optimization of the culture conditions for enhancing *Photorhabdus temperata* biopesticide production using wastewater (WS4) as a raw material. Box-Behnken design (BBD) was used to evaluate the effects of carbon to nitrogen ratio (C/N), sodium chloride concentration and inoculum size on *P. temperata* biomass production and insecticidal activity. For an enhanced biopesticide production, the optimum operating conditions were as follows: inoculum size = 4%; C/N ratio = 12.5 and [NaCl] = 4 g/L for two responses. 1.95 and 2.75 fold improvements in oral toxicity and biomass production were respectively obtained in the cost-effective medium developed in this study (WS4 I) using the three variables at their optimal values. Under the optimized conditions, WS4 I-grown cells exhibited higher membrane integrity according to flow cytometry analysis since dead cells presented only 9.2% compared to 29.2% in WS4. From batch fermentations carried out in WS4 I and WS4, *P. temperata* kinetic parameters in terms of biomass production and substrate consumption rates were modeled. The obtained results showed that the maximum specific growth rate in WS4 I was of 0.43 h^−1^ while that obtained in WS4 was of 0.14 h^−1^. In addition, the efficiency of *P. temperata* to metabolize organic carbon was enhanced by optimizing the culture conditions. It reached 72.66% instead of 46.18% in the control fermentation after 10 h of incubation. Under the optimized conditions, *P. temperata* cells showed the highest specific consumption rate resulting in a toxin synthesis improvement.

## Introduction

Microbial biopesticides based on *Bacillus thuringiensis* are vastly well-known as being a control agent for target pests. However, insects evolving resistance to *B. thuringiensis* delta-endotoxins have been emerged causing a profound threat to subsequent use of these toxins in insect control programs^1^. *Photorhabdus* is a promising alternative for biological control. This entomopathogenic bacterium kills insects through the secretion of a range of toxins including Toxin complexes (Tcs)^2^, *Photorhabdus* insect related (Pir) toxins^3^, Makes caterpillars floppy (Mcf) toxins^4^ and *Photorhabdus* Virulence Cassettes (PVC)^5^. Additionally, *Photorhabdus* produces secondary metabolites which are effective as protein toxins^6^. Through this variety of toxins, this insect pathogenic bacterium is able to infect a broad range of insect hosts belonging to the order of Lepidoptera including *Helicoverpa armigera*, *Spodoptera litura* and *S. exigua*. Indeed, injection of purified Txp40, derived from *P. luminescens* subsp. *akhurstii* to these larvae reduced the number and viability of hemocytes after 12 h of incubation and significantly increased the phenoloxidase activity in the hemolymph leading to melanization reaction and larval death^7^. Moreover, *Plutella xylostella* larvae mortality was observed after oral administration of *P. luminescens*. Mortality rates of *P. xylostella* were of 18.89% and 91.11% after administration of fermentation broth or supernatant, respectively^8^. The Mediterranean flour moth *Ephestia kuehniella* is also a target insect belonging to the order of Lepidoptera. 100% mortality of this insect larvae was reached after oral administration of broth medium at a concentration of 12 × 10^8^ cells/mL^9^. Moreover, mortality of protonymph, deutonymh, adult males and adult females of the polyphagous pest *Tetranychus urticae*, belonging to the order of Trombidiformes was recorded after oral administration of *P. temperata* cell free supernatant^10^. Diptera is also an insect order susceptible to *Photorhabdus* toxins. Indeed, *P. luminescens* suspension had a significant oral toxicity on *Drosophila suzukii* larvae and pupae, with mortalities up to 70–100% after 10 days of treatment^11^. In addition, larvae of *Aedes aegypti* and *Ae. albopictus* belonging to this latter order were demonstrated to be orally susceptible to *P. luminescens* subsp. *akhurstii* with a mortality of 98% after 96 h of treatment^12^.

Although considerable success has been made through the use of *Photorhabdus* as bioinsecticide against different insect orders at a lab scale, the development of low-cost *P. temperata* biopesticide remains a challenge for the industrial production of this bacterium. In this context, a Tunisian industrial wastewater (WS4) has been evaluated for a potential application of low-cost feedstock. However, in this medium, low *P. temperata* biomass production and oral toxicity against *E. kuehniella* were obtained, which were of 4 × 10^8^ cells/mL and 42%, respectively^13^. The improvement of *P. temperata* biomass production was well studied in two media: the optimized medium (OM)^14^ based on glucose and yeast extract and the complex medium (CM)^15^ based on soya bean meal, but it was never reported in wastewater. The enhancement of *P. temperata* cell production is achieved essentially by adding sodium chloride at 5 g/L. Indeed, at such concentration NaCl doubled the biomass production, increased the culturability and the biological activity in both studied media^15^. Moreover, maintaining glucose at 4 g/L in the OM significantly increased *P. temperata* biomass production^16^.

Based on the literature survey, it has been found that C/N ratio and inoculum size greatly influenced the fermentation process in wastes and wastewaters. Indeed, adjustment of C/N at 45 during facultative co-digestion of palm oil mill effluent and empty fruit bunch was demonstrated to enhance methane production^17^. Varying C/N ratio between 7.9 and 9.9 using a combination of sludges increased the entomotoxicity, the growth rate and the viable cell count of *B. thuringiensis*^18^. Inoculum was also identified to affect fermentation in *Agaricus bisporus* wastewater to produce *Saccharomyces cerevisiae* and in distillery wastewater for hydrogen gaz production^19,20^. Moreover, the optimization of inoculum volume during *B. thuringiensis* biopesticide production in waste activated sludge resulted in higher spore, specific growth rate and entomotoxicity values^21^.

Thus, the objective of this study is to identify the optimal conditions for improving the biomass production and the insecticidal activity of *P. temperata* grown in industrial wastewater using Box-Behnken design (BBD). To the best of our knowledge, there are no reports on enhancement of biomass or/and oral toxicity of *P. temperata* using response surface methodology (RSM). Here, NaCl concentration, C/N ratio and inoculum size were taken as three factors of BBD. Biomass production and insecticidal activity were considered as the responses of the system. Additionally, *P. temperata* cell membrane integrity was investigated to track the physiological state during the cell growth in the newly optimized medium comparing to WS4. In the present study, *P. temperata* growth kinetics in both media were also evaluated using mathematical models, which improves our knowledge about *P. temperata* growth behavior and total organic carbon (TOC) consumption efficiency in wastewater.

## Material and methods

### Microorganisms

*Photorhabdus temperata* subsp*. temperata* strain K122 and *P. luminescens* strain Q 167/2 were used in the present work. The K122 strain was used for bioinsecticide production because of its high toxicity to the Lepidopterean insect larvae *E. kuehniella*. *P. luminescens* strain Q 167/2 is a non-pathogenic bacterium, used as a negative control in the bioassay^9^.

### Biopesticide production media

In this study, three media were used: Luria–Bertani (LB) medium, wastewater (WS4) demonstrated to be a suitable medium for *P. temperata* biopesticide production^13^ and the newly optimized medium (WS4 I). WS4 was sampled from the food industry STL (Société Tunisienne de Levure, Beja, Tunisia) and its composition is presented in Table 1. The pHs of the different media were adjusted to 7.0 ± 0.1 before sterilization at 121 °C for 15 min.

**Table 1:** Characteristic of WS4. Data show mean ± standard deviation (n = 3).

Characteristics	WS4
Total solids (TS) (g/L)	7.1 ± 0.11
Volatile solids (VS) (g/L)	4.3 ± 0.22
Suspended solids (SS) (g/L)	0.6 ± 0.05
Volatile suspended solids (VSS) (g/L)	0.6 ± 0.04
pH	7.01
TOC (mg/L)	1828
Total nitrogen (mg/L)	403
C/N ratio	4.53

### Inoculum preparation and growth experiments

One 48 h old colony of *P. temperata* strain K122 was isolated and dispersed into 3 mL of LB medium and incubated overnight at 30 °C. This pre-culture was used to inoculate 500 mL Erlenmeyer flasks containing 85 mL of WS4, with initial optical density of 0.025 at 725 nm^14^. In order to study the effect of inoculum type, a second pre-culture was prepared by inoculating 250 mL Erlenmeyer flask containing 50 mL of WS4 with 1 mL from the first one pre-culture for 10 h of incubation at 30 °C and an agitation of 200 rpm. In this case, different volumes corresponding to different inoculum sizes (1, 2, 3, 4 and 5%) were used to inoculate 500 mL Erlenmeyer flasks. Incubation was carried at the optimized conditions for biopesticide production^14^.

### C/N ratio

As shown in Table 1, WS4 has a C/N ratio of 4.53. As glucose was demonstrated to be an easily assimilated carbon source by *P. temperata* cells^14^, it was selected to adjust the C/N ratio in WS4. This will avoid the difference in the availability of the carbon source brought on by the use of another effluent or waste containing high carbon concentration. In the present work, glucose was added from a stock solution (20%) to obtain a specific C/N ratio varying between 4.53 and 30. This ratio was calculated based on the carbon present in both WS4 and glucose and the nitrogen content in WS4.

### Experimental design and optimization by response surface methodology

To improve *P. temperata* strain K122 biopesticide production in wastewater, an experimental design was developed by RSM. A three-level BBD was used to explore the effects of three independent variables which are: C/N ratio (X_1_), inoculum size (X_2_) and sodium chloride concentration (X_3_) (Table 2). Biomass production and insecticidal activity presented as the total cell count (Y_1_) and the growth inhibition of *E. kuehniella* larvae (Y_2_), respectively were considered as response parameters. The optimization step required 12 experiments and six replicates for the center point which are performed in order to check the validity of the fitted model. Each experiment was done in triplicate and an average value of the response was used for the presentation of the results. The obtained data from BBD were subjected to analysis of variance (ANOVA) to check for errors and the significance of each parameter. Then, data were subjected to a multiple regression analysis to obtain a second-order polynomial regression equation fitted for *P. temperata* biopesticide production (Eq. 1).

1 $$Y = \beta_{0} + \sum\limits_{i = 1} {\beta_{i} x_{i} } + \sum\limits_{i = 1} { \beta_{ii} x_{i}^{2} } + \sum\limits_{i = 1} {\sum\limits_{j = i + 1} {\beta_{ij} x_{i} x_{j} } }$$

**Table 2:** Coded level and real values of independent variables.

Factor	Symbol	Level		
		− 1	0	+ 1
C/N ratio	X1	5	12.5	20
Inoculum size (%)	X2	1	4	7
NaCl (g/L)	X3	2	4	6

where, Y is the predicted response; x_i_ and x_j_ are independent coded variables; β_0_ is an interception coefficient; β_i_, β_ii_ are linear and quadratic regression coefficients, respectively; β_ij_ are regression coefficients of interaction between two variables. Regression analysis, analysis of variance (ANOVA) and response surface plots of the experimental data were performed using the statistical software NEMROD^22^.

To select the effective range of the experimental variables (Table 2), preliminary experiments were conducted with a broad concentration range of NaCl (0.5–10 g/L) and C/N (4.53–30), which were individually supplemented to WS4. Our findings shrink these ranges to (2–6 g/L) and (5–20) shown in Table 2. Besides, using two inocula grown differently, in LB and WS4, the same growth rate and the same biomass production were obtained. Interestingly, a rapid entry in the exponential phase was achieved using WS4 for inoculum preparation (data not shown). Consequently, the use of WS4-grown inoculum as a second step in inoculum preparation was adopted in this study. Likewise, by keeping all other variables at fixed concentrations and varying the inoculum size (0.5–9%), the range of this latter parameter was selected to be from 1 and 7% to design the experimental run.

### Flow cytometry

*Photorhabdus temperata* physiological state study was performed by flow cytometry. WS4 and WS4 I-grown cells were sampled at two incubation times (24 h and 48 h). Fresh-cells were diluted with PBS 1 × pH 7.2 to a final concentration of 10^6^ cells/mL then stained with propidium iodide (PI) at a final concentration of 10 μg/mL followed by an incubation period of 15 min in the dark. *P. temperata-*heated cells at 70 °C for 15 min were used as a positive control^13^. Flow cytometry analysis was carried out using Attune Nxt Acoustic Focusing Flow Cytometer (Thermo fisher) equipped with a 488 argon laser. Fluorescent beads of 2 µm in diameter (Fluoresbrite, Polyscience) were added in order to normalize the flow cytometer settings. For each sample data were collected for 10,000 events, using logarithmic amplification, at a flow rate of 100 μL/min. *P. temperata* populations were defined using the region gates based on FSC (forward scatter) and SSC (side scatter) correlated to the cell size and to the cell granularity, respectively. Gated population was further represented in a bivariate dot plot according to the PI emission, collected at 695 ± 40 nm band pass filter (BL3), on the ordinate axis and to the high angle SSC on the abscissa axis. For each sample assay was run in duplicate.

### Analytical procedures

*Photorhabdus temperata* fermentations were carried out in 500 mL Erlenmeyer flasks containing 85 mL of WS4 and WS4 I. Incubation was performed at 30 °C in a rotary shaker set at 200 rpm during 48 h^14^. For RSM study, samples were collected at the end of fermentation and were subjected to determination of total cell count and insecticidal activity as reported by Jallouli et al.^9,14^, respectively. 48 h of incubation corresponds to the maximal biomass production and toxin synthesis^9,14^. Total direct count was microscopically determined using Thoma counting chamber at 100-fold magnification. For this purpose, samples were diluted in order to have a maximum of 10 to 15 cells and a minimum of three cells per mm^2^^14^. Bioassays were carried out using ten *E. kuehniella* larvae which were weighed before they were transferred to a sterile Petri dish containing 1 g of wheat flour mixed with 800 µL of the fermentation broth at a cell density of 4 × 10^8^ cells/mL. Then, the weight of the ten larvae was recorded after incubation at 26 °C for 7 days. Insecticidal activity was assessed as the growth inhibition of the fed *E. kuehniella* larvae with K122, compared to the growth of similar larvae number fed with the non-toxic *P. luminescens* strain Q cultured at the same conditions^9^. The growth inhibition was calculated as showing in Eq. (2):

2 $${\text{Growth inhibition }}\left( {\text{\% }} \right) = { }\left( {\frac{{{\text{GQ}} - {\text{GK}}122}}{{{\text{GQ}}}}} \right) \times 100$$

GQ: (weight of the ten larvae fed with strain Q after 7 days) − (weight of the ten larvae fed with strain Q at t = 0).

GK122: (weight of the ten larvae fed with strain K122 after 7 days) − (weight of the ten larvae fed with strain K122 at t = 0).

To compare the relationship between insecticidal activity and fermentation time during growth in WS4 and WS4 I, the same bioassay was performed using the same cell count of 4 × 10^8^ cells/mL after 24 h, 30 h and 48 h of incubation. For kinetic study, *P. temperata* biomass production and substrate concentration during fermentation were determined every hour during 30 h. Biomass concentration on a dry basis (total solids (TS)) was gravimetrically determined. Samples were periodically taken from the fermentation broth, centrifuged (13,000 rpm for 5 min) at room temperature, washed twice with saline water (9%) and dried at 105 °C in pre-weighed porcelain vials until constant weight^23^. The total solids (TS) content of WS4 and WS4 I before inoculation was subtracted from all TS samples to obtain the TS equivalent to biomass production at each incubation time. The substrate concentration during fermentation in WS4 and WS4 I was quantified through the determination of TOC concentration. Total organic carbon (TOC) is determined by dry combustion at high temperature and the CO_2_ released is detected by means of an infrared sensor using Shimadzu TOC analyzer TOC-VCPH according to standard methods^23^.

### Mathematical modeling

As growth substrates (carbohydrate and nitrogen) present in wastewater were considered to be in excess during the batch fermentations, the exponential growth rate could be expressed as first order equation. Thus, *P. temperata* kinetic parameters (r_X_, r_S_, µ, µ_max_ and q_S_) could be determined from mathematical models illustrated in equations from **3 to 6**. In this study, production rate of K122 toxins was not estimated because, until now, there is no method allowing toxin quantification.

3 $$\frac{dX}{{dt}} = rx = \mu X$$

4 $$\frac{dS}{{dt}} = - rs$$

5 $$\frac{rx}{X} = \mu$$

6 $$\frac{rs}{X} = - qs$$

### Statistical analysis

A logistic model (LIS Excel) in Microsoft Excel software (version 2007, Microsoft Corporation) was used to calculate *P. temperata* kinetic parameters and to adjust the obtained results. GraphPad Prism 7 software (version 7.04; www.graphpad.com) was employed to design kinetic figures. For RSM model development, regression analysis and analysis of variance (ANOVA), NEMROD statistical software (Logiciel Nemrod-W, LPRAI, Marseille, France, version 2000-D; www.Nemrodw.com) was used. All results related to the determination of TS, TOC concentration and bioassays were the average of three replicates of three separate experiments. They were statistically analyzed by SAS software (Version 6) using Student’s test performed after analysis of variance (ANOVA). Values were considered significantly different with *p* < 0.05, *p* < 0.01 (*), *p* < 0.001 (**), or *p* < 0.0001 (***).

## Results and discussion

### Response surface methodology: Box-Behnken design

In this study, we tried to analyze model and interpret the experimental data using RSM as a mathematical modeling system. In this regard, a twelve-run BBD design with three levels and three factors with six replications at the central point was designed to study the optimum combination of NaCl concentration, C/N ratio and inoculum size for maximum biomass production and insecticidal activity of the insect pathogenic bacterium *P. temperata*. The experimental designs as well as the experimental results are presented in Table 3. The analysis of variance (ANOVA) of the response surface quadratic model for biomass production and growth inhibition of *E. kuehniella* larvae was presented in Table 4. The obtained results showed that *p*-values reveal significance for both regression models (*p* ˂ 0.0001). Moreover, according to Table 4, the lack of fit is not significant for both responses (*p* ˃ 0.05). Consequently, both models could predict the optimal biomass production and insecticidal activity and define optimal variable values. As shown in Table 5, the coefficients of determination (R^2^) were of 0.989 and 0.991, for biomass and oral toxicity responses, respectively. This indicates that 98.9% and 99.1% of the variability in the response could be explained by the model which reflects a good correlation between experimental and predicted values. The adjusted coefficient of determination values (Adj R^2^ = 0.976 and 0.981, respectively) were within reasonable agreement with predicted R^2^.

**Table 3:** Details of experimental attempts used in the response surface methodology (RSM) optimization.

Run	Coded levels			Results	
	C/N ratio X_1_	Inoclum size (%) X_2_	NaCl (g/L) X_3_	Total cell counts (10^8^ cells/mL)	Insecticidal activity (%)
**1**	− 1	− 1	0	5.00	40.40
**2**	1	− 1	0	4.00	37.84
**3**	− 1	1	0	9.30	56.13
**4**	1	1	0	9.50	81.66
**5**	− 1	0	− 1	6.00	37.40
**6**	1	0	− 1	7.80	47.79
**7**	− 1	0	1	9.75	47.34
**8**	1	0	1	10.00	60.44
**9**	0	− 1	− 1	2.00	15.34
**10**	0	1	− 1	6.00	50.18
**11**	0	− 1	1	4.75	30.30
**12**	0	1	1	10.00	70.34
**13**	0	0	0	10.50	80.16
**14**	0	0	0	10.75	83.79
**15**	0	0	0	10.75	80.80
**16**	0	0	0	10.50	77.90
**17**	0	0	0	10.50	78.87
**18**	0	0	0	10.00	80.14

**Table 4:** ANOVA for the quadratic model.

Source	Total cell count (10^8^cells/mL)					Insecticidal activity (%)				
	SS	DF	MS	F	P	SS	DF	MS	F	P
Model	130.43	9	14.49	76.88	< 0.0001***	7.64	9	8.49	98.07	< 0.0001***
Residual	1.50	8	0.18			6.93	8	8.66		
Lack of fit	1.13	3	0.37	5.03	0.058	4.90	3	1.63	4.03	0.084
Pure error	0.37	5	0.07			2.02	5	4.05		
Total	131.94	17				7.71	17			

*SS* sum of squares, *DF* degree of freedom, *MS* mean square, *F* F-value, *P* P-value.

***Significant at the level 99.9%.

**Table 5:** Estimated regression coefficients corresponding to the Box-Behnken design.

Name	Total cell count (10^8^cells/mL)				Insecticidal activity (%)			
	Coefficient	Error	t.exp	Significant	Coefficient	Error	t.exp	Significant
b 0	10.500	0.177	59.24	< 0.0001***	80.28	1.20	66.80	< 0.0001***
b 1	0.156	0.154	1.02	0.34	5.81	1.04	5.58	0.0006
b 2	2.381	0.154	15.51	< 0.0001***	16.80	1.04	16.15	< 0.0001***
b 3	1.587	0.154	10.34	< 0.0001***	7.21	1.04	6.93	0.0001***
b 11	− 0.425	0.208	− 2.04	0.073	− 9.78	1.41	− 6.94	0.0001***
b 22	− 3.125	0.208	− 15.03	< 0.0001***	− 16.49	1.41	− 11.70	< 0.0001***
b 33	− 1.688	0.208	− 8.12	< 0.0001***	− 22.25	1.41	− 15.79	< 0.0001***
b 12	0.300	0.217	1.38	0.20	7.02	1.47	4.77	0.0015**
b 13	− 0.388	0.217	− 1.78	0.11	0.68	1.47	0.46	0.66
b 23	0.313	0.217	1.44	0.18	1.30	1.47	0.88	0.40

R^2^ (Y_1_) = 0.989; Adj R^2^ (Y_1_) = 0.976; R^2^ (Y_2_) = 0.991; Adj R^2^ (Y_2_) = 0.981.

***Significant at the level 99.9%.

**Significant at the level 99%.

The second order polynomial regression equation fitted into the experimental data for total cell count response (Y_1_) is as follows (Eq. 7):

7 $${\text{Y}}1{ } = { }10.5{ } + { }0.16{\text{ X}}_{1} + { }2.38{\text{ X}}_{2} + { }1.59{\text{ X}}_{3} { }{-}{ }0.43{ }\left( {{\text{X}}_{1}^{2} } \right){ }{-}{ }3.125{ }\left( {{\text{X}}_{2}^{2} } \right){ }{-}{ }1.69{ }\left( {{\text{X}}_{3}^{2} } \right) + { }0.30{ }\left( {{\text{X}}_{1} {\text{ X}}_{2} } \right){ }{-}{ }0.39{ }\left( {{\text{X}}_{1} {\text{ X}}_{3} } \right) + { }0.31{ }\left( {{\text{X}}_{2} {\text{ X}}_{3} } \right)$$

The significance of each coefficient, determined by *p*-values, is summarized in Table 5. The *p*-values imply that the first and second order main effects of X_2_ and X_3_ are significant (*p* ˂ 0.0001). However, none of the interaction effects are significant (*p* ˃ 0.05). Moreover, the fitted equation for prediction of *P. temperata* cell toxicity (Y_2_) is as follows (Eq. 8):

8 $${\text{Y}}2{ } = { }80.28{ } + { }5.81{\text{ X}}_{1} { } + { }16.80{\text{ X}}_{2} { } + { }7.21{\text{ X}}_{3} { }{-}{ }9.78{ }\left( {{\text{X}}_{1}^{2} } \right){ }{-}{ }16.49{ }\left( {{\text{X}}_{2}^{2} } \right){ }{-}{ }22.25{ }\left( {{\text{X}}_{3}^{2} } \right){ } + { }7.02{ }\left( {{\text{X}}_{1} {\text{ X}}_{2} } \right){ } + { }0.68{ }\left( {{\text{X}}_{1} {\text{ X}}_{3} } \right){ } + { }1.30{ }\left( {{\text{X}}_{2} {\text{ X}}_{3} } \right)$$

As shown in Table 5, the first and second order main effects of X_2_ and X_3_ and the second order of X_1_ are found to be significant (*p* ≤ 0.0001), as well as the interaction effect between X_1_ and X_2_ (*p* = 0.0015).

**Figure 1:**
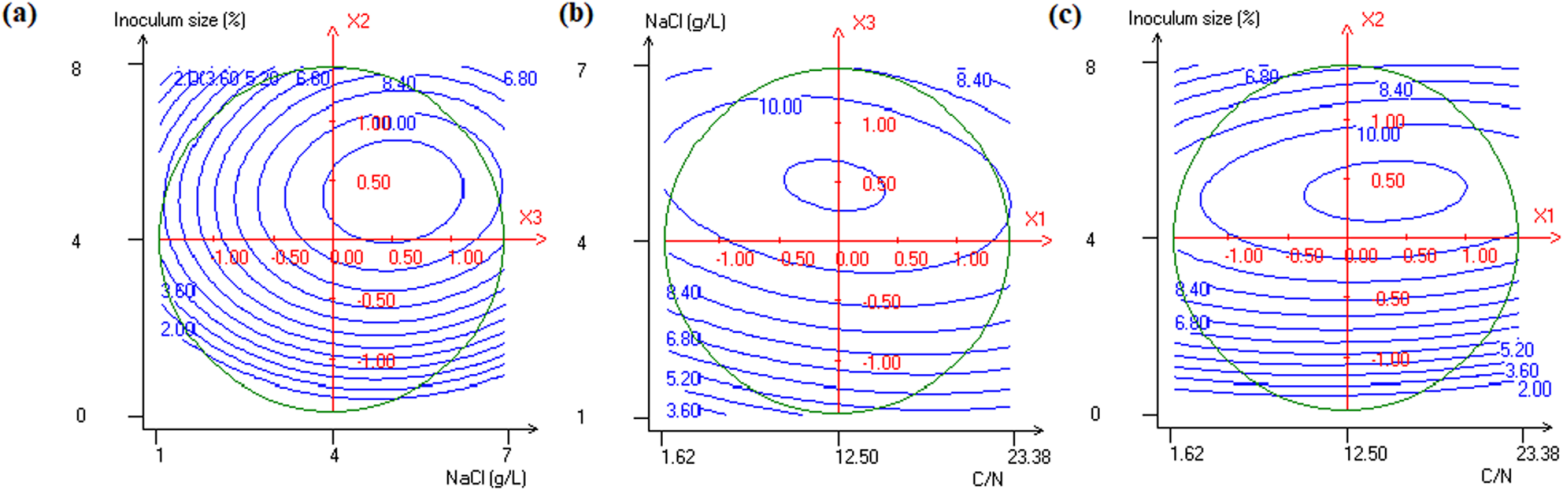
Contour plots of biomass production showing interactive effects of **(a)** inoculum size and NaCl, **(b)** NaCl and C/N, **(c)** inoculum size and C/N. The third parameter is at his central value.

**Figure 2:**
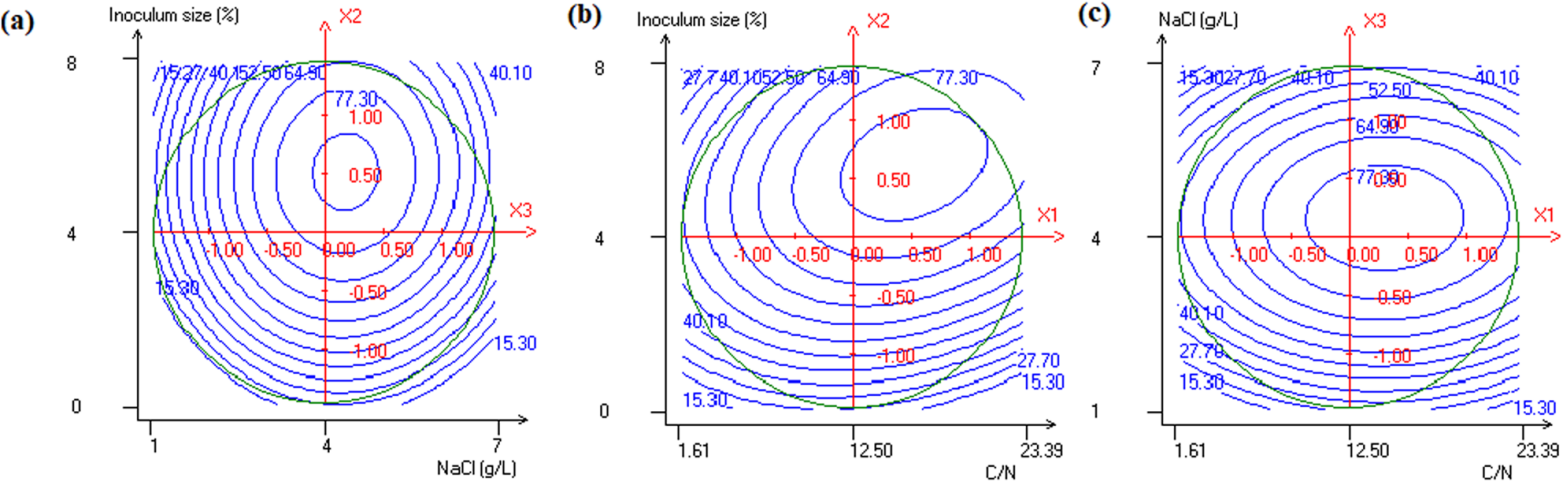
Contour plots of insecticidal activity showing interactive effects of **(a)** inoculum size and NaCl, **(b)** inoculum size and C/N, **(c)** NaCl and C/N. The third parameter is at his central value.

Surface and contour plots generated by the software NEMROD are presented in Figs. 1 and 2. These figures were plotted to examine the relationship between the different paired factors and to determine the optimum of each one for the highest biomass production and insecticidal activity. As shown in Fig. 1a, biomass production increases with the increase of inoculum size and sodium chloride concentration to reach a maximum value of 11.4 × 10^8^ cells/mL obtained at range of 4–6.5% and 3.8–6 g/L, respectively. Similarly, previous studies reported that the addition of sodium chloride at 5 g/L to the OM and the CM doubles the biomass production of the strain K122 of *P. temperata*. In fact, NaCl was demonstrated to be a stimulator of growth of the strain K122 by increasing nutrient assimilation^15^. Moreover, sodium chloride is involved in stimulation of the uptake of compatible solutes involved in *Photorhabdus* cell protection and growth rate increase^24,25^. It was also reported that increase of *P. temperata* inoculum size causes improvement of biomass production^16^. Indeed, by increasing inoculum size from 0.05 to 0.15 optical density unit, biomass increased both in LB medium and the OM. Moreover, Lachhab et al.^21^ showed improvements of *B. thuringiensis* total viable cell and spore counts by varying inoculum size from 2 to 4%. However, increasing inoculum size of *P. temperata* above 6.5%, at optimal NaCl concentration of 4 g/L, has a negative effect on biomass production (Fig. 1a). Inhibition of *P. temperata* cell growth could be explained by the fact that high initial K122 cell concentration resulted in a rapid consumption of oxygen and nutrients resulting in a low final biomass production. By plotting NaCl concentration or inoculum size against C/N ratio (Fig. 1b,c), the obtained results showed that C/N ratio seems to have no effect on biomass production when inoculum size and NaCl concentration were at low levels. In contrast, when WS4 was supplemented by an inoculum volume and a sodium chloride concentration upper than 4.5% and 4 g/L, respectively, a high biomass production was obtained (11.4 × 10^8^ cells/mL). This high level was reached only when adjusting the C/N ratio to a range of 9–15 (Fig. 1b) and a range of 9.8–20 (Fig. 1c). Thus, it is evident that keeping a balanced composition of C/N in wastewater, by adding available carbon source, is required for improving the total cell production. Indeed, in fermentation C/N ratio is more important than the nitrogen concentration for increasing the cell density and the desired product concentration^26^. It has been suggested that C/N ratio affects the expression of tricarboxylic acid (TCA) cycle genes affecting by-products accumulation which, in turn, disrupts cell growth^27^. It is well known that the primary form of *Photorhabdus* produced different typical by-products such as acetate, lactate and formate during carbohydrate metabolism^28^. Thus, the unbalanced nutrient status in WS4 could be responsible for the accumulation of such forms decreasing *P. temperata* biomass production. In agreement with this, Shiloach and Rinas^29^ reported that acetate accumulation during carbohydrate assimilation is considered an obstacle to the enhancement of *Escherichia coli* bacterial growth. Moreover, Wisuthiphaet and Napathorn^30^ reported that using an optimal C/N ratio when culturing *Azohydromonas lata* on various cane sugar products improved its growth rate and productivity.

To illustrate the interaction effect between inoculum size, NaCl concentration and C/N ratio for maximum insecticidal activity, contour plots were drawn (Fig. 2). The obtained results showed that the maximum toxin synthesis occurred when increasing inoculum size and sodium chloride concentration beyond 4% and 3.5 g/L, respectively (Fig. 2a). The enhancement of *P. temperata* toxin synthesis through NaCl addition was demonstrated by Jallouli et al.^15^. Indeed, an improvement of 81.4 and 42.22% of *P. temperata* oral toxicity in the CM and the OM, respectively, was obtained when NaCl was added beyond 2 g/L. An improvement of *B. thuringiensis* delta-endotoxin production was also reported when NaCl was added at 7 g/L^31^. Moreover, the present work demonstrates for the first time the involvement of inoculum size and C/N ratio in K122 toxin synthesis. Indeed, according to Fig. 2b, these variables have a positive effect on *P. temperata* toxicity. The highest toxicity of 85% was obtained using an inoculum volume of 5.5% and a C/N ratio of 12.5, respectively. At an optimal C/N value of 12.5, exceeding inoculum size range between 4.3 and 7%, reduced considerably *P. temperata* toxicity. This fact could be due to the low biomass production obtained by using high and low initial cell concentrations resulting in a decrease of the final entomotoxicity. Similar findings were reported by Lachhab et al.^21^ when studying *B. thuringiensis* entomotoxicity in wastewater sludge. It is particularly important to note that the lowest toxicity of 15.3% was obtained at (− 1) and (+ 1) levels of C/N along with (− 1) and (+ 1) levels of NaCl concentration (Fig. 2c)*.* This could be explained by the fact that at high and low levels there is a decline in *P. temperata* structural metabolism affecting K122 toxin gene expression. Indeed, protein synthesis is the most energy consuming process among all anabolic activities that might be limited essentially by carbon and nitrogen flux alteration in *P. temperata* metabolic pathway. Interestingly, by adjusting the C/N ratio, the strain K122 could use glucose as an energy source leading to the generation of the ATP required for the biosynthetic metabolism. At the same time, this bacterium could overcome by-product accumulation involved in the inhibition of metabolite synthesis. These results were in agreement with those reported by Vidyarthi et al.^18^ demonstrating that it is necessary to optimize the C/N ratio during *B. thuringiensis* fermentation in sludge to enhance its entomotoxicity. Further, Wang et al.^32^ and Shiloach and Rinas^29^ stated that the optimization of C/N ratio enhanced polymer productivity and recombinant protein production by activated sludge bacteria and *E. coli*, respectively. Therefore, from the optimization plots, the maximum response of biomass production and insecticidal activity occurred at an inoculum size of 4%, a NaCl concentration of 4 g/L and a C/N ratio of 12.5. At these conditions, the total cell count and the oral toxicity were of 11.4 × 10^8^ cells/mL and 85%, respectively, which correspond to an improvement by 185 and 102.38%, respectively, compared to WS4. This finding is interesting from a practical point of view for the production of low-cost *P. temperata* bioinsecticide. In fact, the cost of WS4 I medium developed herein is limited to 35 US$ per kilogram, compared to the OM^14^ whose price have been estimated to be up to 679 US$ per kilogram, which represents a reduction by almost 94.84% of the overall production cost. To experimentally validate the predicted response, *P. temperata* fermentation was carried out using the newly optimized medium WS4 I. The validation experiment carried out under the optimized conditions showed that the experimentally determined biomass production value (11 × 10^8^ cells/mL) and the oral toxicity (82%) were in agreement with the statistically predicted ones (11.4 × 10^8^ cells/mL and 85%), confirming the model’s authenticity.

### Cell physiology study by flow cytometry

**Figure 3:**
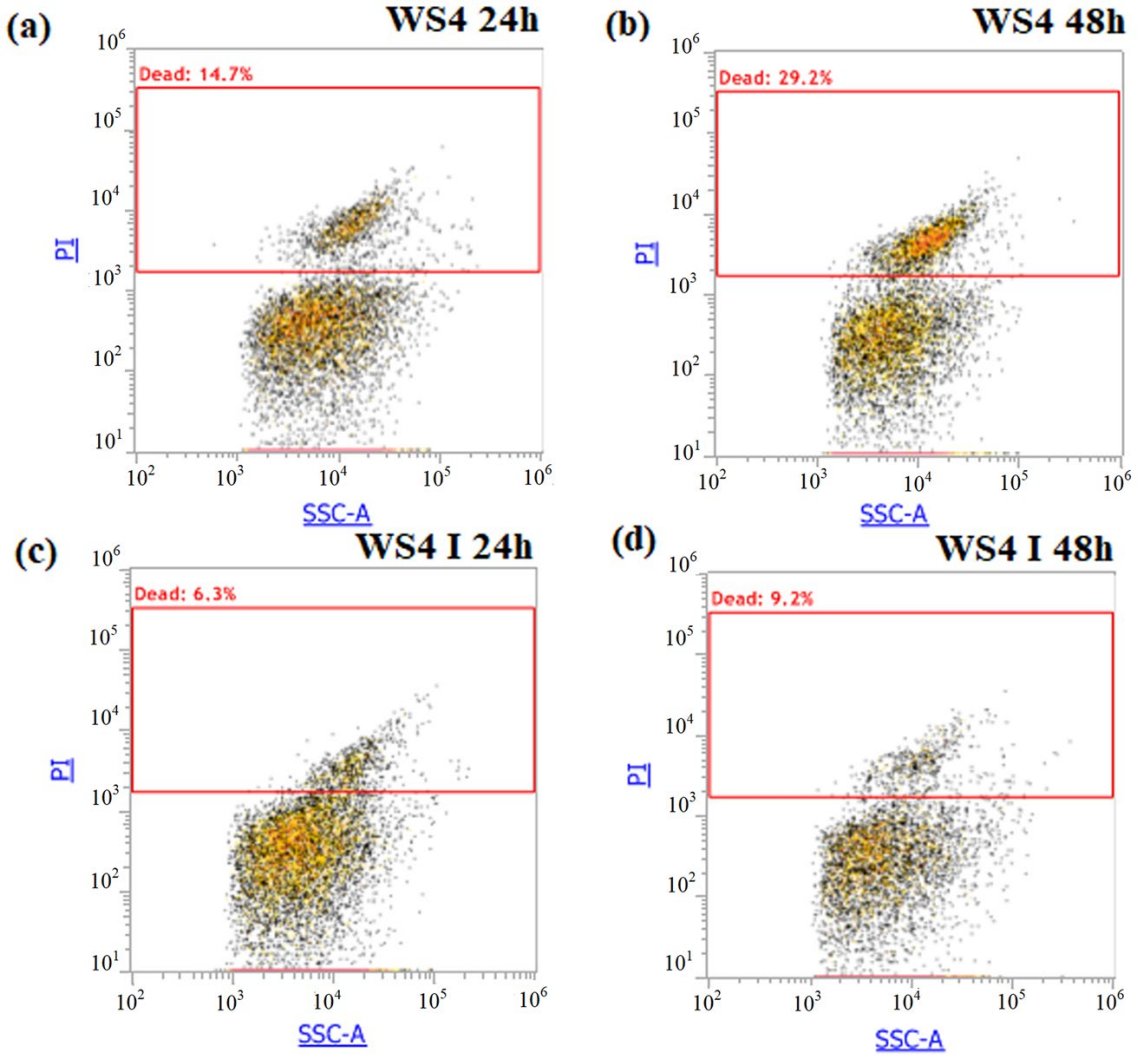
PI single stained dot plots of *P. temperata:* **(a)** WS4 at 24 h; **(b)** WS4 at 48 h; **(c)** WS4 I at 24 h and **(d)** WS4 I at 48 h.

To compare the physiological state of the strain K122 cultured separately in WS4 and WS4 I, flow cytometry analysis was carried at 24 h and 48 h of incubation (Fig. 3). According to the PI single stained dot plots, 14.7% of K122 PI positively stained cells appear since 24 h in WS4. This level increased to 29.2% after 48 h of incubation indicating the loss of membrane integrity after prolonged incubation in wastewater (Fig. 3a). These data are consistent with those reported by Keskes et al.^13^ reporting enhancement of *P. temperata* cell death after prolonged incubation in different industrial wastewaters. However, here the percentage of WS4-PI positively stained cells increased compared to these reported results^13^. This could be explained by the variation in the chemical and physical composition of this effluent influencing accumulation of reactive oxygen species. Production of such by-products during *P. temperata* cell metabolism has been considered as a key factor triggering its cell death^33^. Interestingly, by culturing *P. temperata* in the newly optimized medium, viability increased since dead cell represents only 6.3% and 9.2% of the total existing cells after 24 h and 48 h of incubation, respectively (Fig. 3b). Differences in the physiological state of the strain K122 between the studied media could be attributed to the level of ROS accumulation during assimilation of the OM in WS4 or the OM mixed with glucose in WS4I as suggested by Xiao et al.^34^ showing differences in ROS concentration using different carbon sources during *Pichia pastoris* fermentation. Thus, at the optimized conditions, WS4 I-grown cells exhibited a metabolism pathway that avoids by-product accumulation and particularly ROS generation. Moreover, these findings could be explained by the variation in ROS buffering ability of K122 cells cultured in WS4 and WS4 I. Differences in cells resistance to ROS accumulation and scavenging has been demonstrated in *S. cerevisiae* wine strain during fermentation of high-sugar-containing medium and has been also shown to be affected by medium composition^35,36^.

### Determination of specific rates of cell growth and substrate consumption

**Figure 4:**
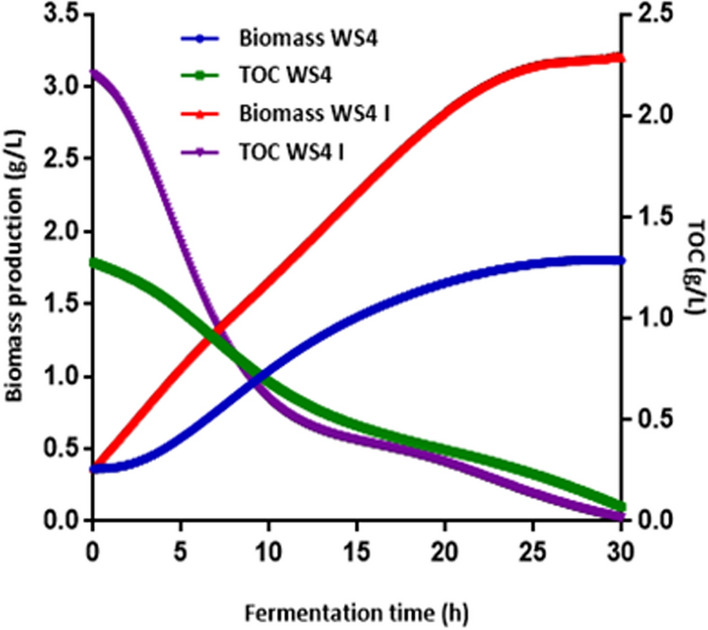
Biomass production and TOC concentration profiles during *P. temperata* fermentation in WS4 and WS4 I.

**Figure 5:**
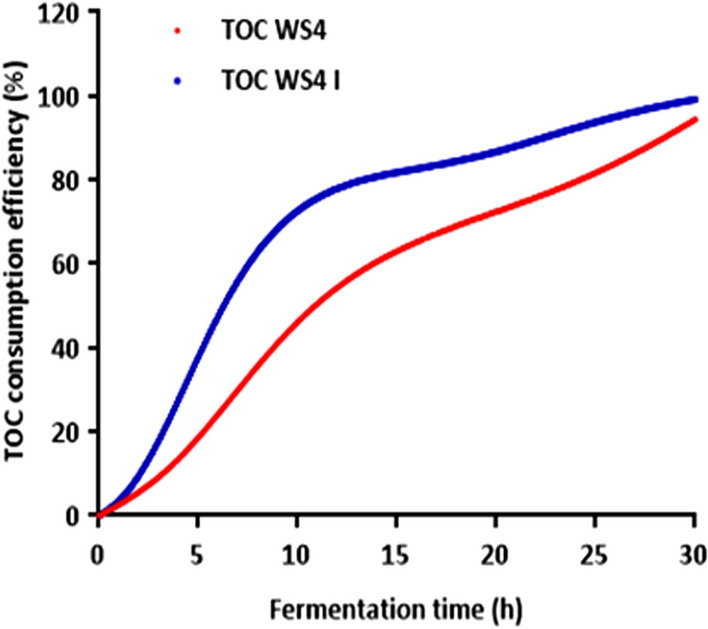
TOC consumption efficiency during *P. temperata* fermentation in WS4 and WS4 I.

To achieve a modeling for large scale *P. temperata* biopesticide production in industrial wastewater, kinetic models that relate consumption of total organic carbon and biomass production during batch fermentation are required. At our knowledge, this is the first report in literature focusing on the mathematical modeling of *P. temperata* growing in wastewater as a low-cost feedstock. Figure 4 shows growth curves and substrate consumption in optimized medium WS4 I and WS4 considered as control. From the obtained results, it is clear that *P. temperata* growth was enhanced by optimizing the growth conditions (NaCl concentration, inoculum volume and C/N ratio). Indeed, biomass concentration in WS4 significantly increased (p < 0.05) from 0.49 g/L obtained at 4 h of incubation to 1.75 g/L after 24 h of incubation. However, in the RSM optimized medium the biomass production started to increase from one hour reaching a high value of 3.1 g/L after the same incubation time. Thus, using the optimized conditions for *P. temperata* biopesticide production in batch fermentation reduced the lag phase by three hours and increased the final biomass production while maintaining 24 h as the time at which *P. temperata* enters into the stationary phase. Additional results from Fig. 4, analyzed according to Student’s test performed after ANOVA, showed that total organic carbon concentration significantly decreased from 1.8 g/L and 2.2 g/L at inoculation to 0.1 g/L and 0.018 g/L (p < 0.01) after 30 h of fermentation in WS4 and WS4 I, respectively. This indicates a TOC consumption efficiency of 94.4% and 99.2%, respectively, after 30 h of incubation. The slight increase in initial TOC level in the optimized medium is due to glucose addition for adjusting the C/N ratio at 12.5. The increase in TOC consumption efficiency could be confirmed from results illustrated in Fig. 5. Indeed, by plotting TOC consumption efficiency versus fermentation time, similar curves were obtained but with a low slope value of 5.42 in the control fermentation, compared to 9.29 obtained in the RSM optimized medium. The similar trends observed in WS4 and WS4 I indicate that the strain K122 was able to use the organic matter together with glucose, as an easily degradable substrate, showing that the synthesis of enzymes involved in organic matter biodegradation is not influenced by the presence of high extracellular glucose concentrations. This is consistent with the work of Bally and Egli^37^ reporting the same observation when culturing *Chelatobacter heintzii* on nitrilotriacetate (NTA) or on a mixture of NTA and glucose. Furthermore, the presence of glucose in WS4 I, supplied for adjusting the C/N ratio, have a strong influence on the catabolic activity of the strain K122 illustrated through the improvement of TOC consumption efficiency (Fig. 5). In fact, the added-glucose could avoid by-product accumulation and provide the energy necessary for *P. temperata* cellular metabolism and particularly for synthesis of organic matter degrading enzymes. At these conditions, WS4 I grown-cells were not forced to use their own constituents as an energy source, which contributes in lag phase reduction as observed in Fig. 4. Hence, the obtained results suggest that TOC consumption efficiency is highly dependent on the rate of enzyme biodegradation expression which is accelerated in WS4 I due to glucose addition. Moreover, the enhancement of nutrient assimilation by the strain K122 could be also explained by sodium chloride addition at 4 g/L in WS4 I as demonstrated for the CM and the OM used for *P. temperata* bioinsecticide production^15^.

**Figure 6:**
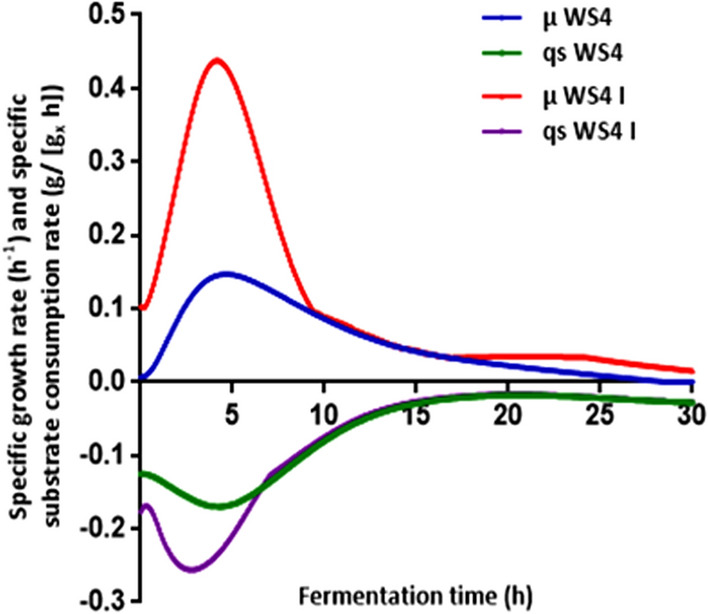
Specific growth rate (µ) and specific substrate consumption rate (q_S_) during *P. temperata* fermentation in WS4 and WS4 I.

Comparison between the specific growth and specific substrate consumption rates for WS4 and WS4 I is presented in Fig. 6. The close association between *P. temperata* cell growth and substrate consumption was observed from the variations of the specific growth rate (µ) and the specific substrate consumption rate (q_S_) in these two fermentation processes. In general, an increase of the specific growth rate was followed by a decrease of the specific consumption rate and vice versa. As shown in Fig. 6, the variation in the specific growth rate in WS4 and WS4 I followed similar patterns with a µ_max_ of 0.14 h^−1^ (R^2^: 0.99) (p ˂ 0.001) and 0.43 h^−1^ (R^2^: 0.99) (p ˂ 0.001) at 4 h of incubation, corresponding to 4.95 h and 1.61 h of doubling time (dt), respectively. This fact is interesting from a practical point of view for the production of *P. temperata* bioinsecticide in low-cost feedstock medium. The obtained value of µ_max_ is different from that presented in a previous report on *P. luminescens* indicating a maximum specific growth rate varying between 0.36 h^−1^ and 0.33 h^−1^ in media based on nutrient broth and complex nutrients, respectively^38^. Further, the obtained specific growth rates differ from the ones previously reported (μ = 0.36 h^−1^, dt = 2.1 h) for *P. luminescens* in nutrient broth^39^ and those (0.21 h^−1^, dt = 3.3 h) obtained by culturing *P. luminescens* subsp. *akhurstii* SL0708 in medium based on 10 g/L yeast extract and 3 g/L glucose^40^. Likewise, O’Campo et al.^41^ reported that using Yoo medium for *P. luminescens* growth, a specific growth rate of 1.4 h^−1^ was obtained corresponding to doubling time of 0.51 h. These variations could be attributed to bacterial isolates used and also to different media composition and culture conditions. Thus, it is evident that under the optimized conditions the growth rate significantly increased with P > F value ˂ 0.001, throughout the fermentation giving higher *P. temperata* biomass production.

**Figure 7:**
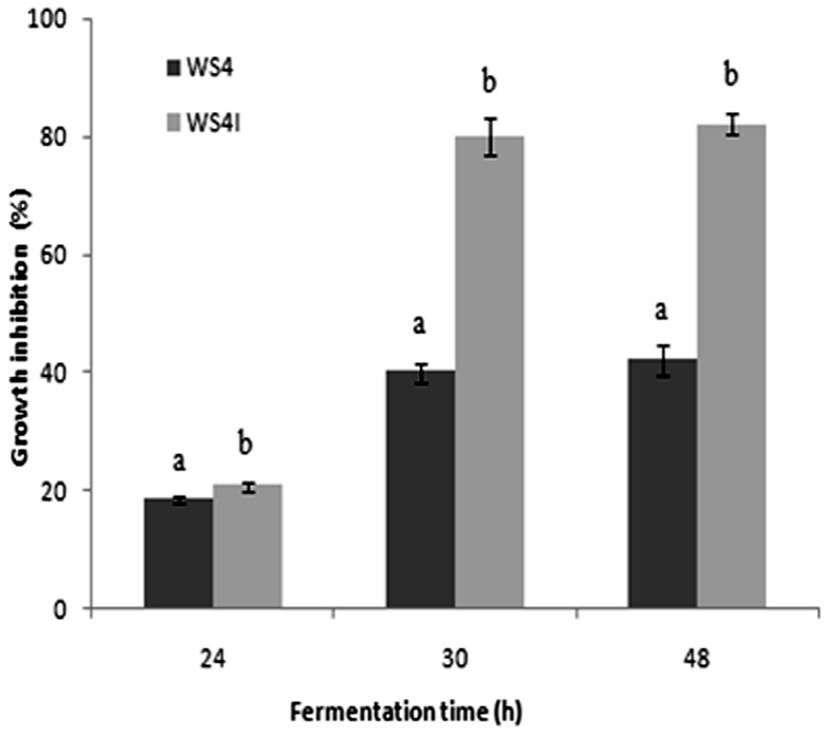
Effect of the production medium on *P. temperata* K122 oral toxicity. Letters (**a**,**b**) above each column indicate significant differences among oral toxicity of the strain K122, cultured in WS4 and WS 4 I at *p* < 0.05.

The Student’s test with a low probability value [(P > F value) ˂ 0.001] indicated that the specific consumption rate of substrate was enhanced in WS4 I with a maximum value of 0.25 (g/[g_X_ h]) compared to 0.16 obtained in WS4 (Fig. 6) confirming results obtained in Fig. 5. This is expected to have a positive effect on the specific metabolite production rate^42^. Indeed, assessment of oral toxicity against *E. kuehniella* larvae after different times of incubation in the studied media at the same cell density of 4 × 10^8^ cells/mL, showed that *P. temperata* cultured cells in WS4 I medium exhibited a higher toxicity of 20.8 (p ˂ 0.05) and 82% (p ˂ 0.0001) at 24 h and 48 h, respectively, than those produced in WS4 showing an inhibition growth of *E. kuehniella* larvae of 18.5 (p ˂ 0.05) and 42% (p ˂ 0.0001) after the same incubation times (Fig. 7). Increase in *P. temperata* toxicity highlights that the change in *P. temperata* behavior during fermentation in WS4 I contributes to the overcome of by-product accumulation and/or the provision of energy likely to enhance the toxin production.

## Conclusion

The present research indicates that RSM was effective to optimize a combination of factors influencing *P. temperata* bioinsecticide production. The designed medium was useful for producing high biomass production, viability and insecticidal activity which were of 11 × 10^8^ cells/mL, 90.8 and 82%, respectively, after 48 h of incubation. Under the optimized conditions, the specific growth and the substrate consumption rates were improved leading to an improvement of *P. temperata* toxin synthesis. Thus, enhancing biopesticide production in wastewater is a promising strategy to achieve a low-cost and a high active toxin production for large-scale application.

## Abbreviations

*P. temperata*: *Photorhabdus temperata*
*B. thuringiensis*: *Bacillus thuringiensis*
WS4: Industrial wastewater
WS4 I: The newly optimized medium
CM: Complex medium
OM: Optimized medium
Tcs: Toxin complexes
Pir toxins: *Photorhabdus* insect related toxins
Mcf toxins: Makes caterpillars floppy toxins
PVC: *Photorhabdus* Virulence Cassettes
*S. exigua*: *Spodoptera exigua*
*P. xylostella*: *Plutella xylostella*
*E. kuehniella*: *Ephestia kuehniella*
*Ae. Albopictus*: *Aedes albopictus*
BBD: Box-Behnken design
RSM: Response surface methodology
TOC: Total organic carbon
TS: Total solids
TCA: Tricarboxylic acid
PI: Propidium iodide
*S. cerevisiae*: *Saccharomyces cerevisiae*

## Nomenclature

µ: Specific growth rate (h^−1^)
µ_max_: Maximum specific growth rate (h^−1^)
q_S_: Specific substrate consumption rate (g/[g_X_ h])
r_S_: Substrate consumption rate (g/[L h]);
r_X_: Biomass production rate (g_X_/[L h])
S: Substrate concentration (mg/L)
X: Biomass concentration (g_X_/L)
t: Time (h)
